# The 4Ms in a Pandemic: A Survey of Training Needs Among Healthcare Professionals, Older Adults, and Community Members

**DOI:** 10.1093/geroni/igab046.3019

**Published:** 2021-12-17

**Authors:** Jennifer Crittenden, Abigail Elwell, David Wihry, Lenard Kaye

**Affiliations:** 1 University of Maine, University of Maine, Maine, United States; 2 University of Maine, Bangor, Maine, United States

## Abstract

The University of New England, in collaboration with the University of Maine, received a five-year grant from the Health Resources and Services Administration (HRSA) to improve the health and well-being of Maine’s older adults through enhanced training under HRSA’s Geriatrics Workforce Enhancement Program (GWEP). As part of these efforts, stakeholder training needs assessment data were collected via a statewide electronic survey that was distributed to community members and providers throughout Maine. The survey, which focused on the 4M’s of Age-Friendly Healthcare, received 68 responses from older adults/community members (N = 26), program administrators (N = 12), along with community leaders, and those working in the public and non-profit sector (N = 13). A significant emphasis on social isolation, mental health, and grief and loss issues was noted and dominating themes centering on two dimensions of the 4M framework: “What Matters” and “Mentation.” Findings reflect an overriding priority by providers and consumers to keep older adults socially connected (28%, N = 34) and maintaining mental health and well-being during the pandemic (21%, N = 14). Qualitative response analysis identified additional COVID-19-related training topics such as: what to do if you or a loved one contracts coronavirus, how to handle grief and loss related to COVID-19, strategies for supporting loved ones during COVID-19, and socially distanced bereavement support. Results indicate a need to focus on meeting the emotional and mental health needs of older adults, as well as the importance of encouraging connections and mitigating the effects of social isolation during COVID-19.

